# Abdominal Etching—A Novel Classification Method for Surgical Approach

**DOI:** 10.1007/s00266-024-04384-5

**Published:** 2024-10-01

**Authors:** Avraham Levy, Ariel Berl, Ofir Shir-az, Din Mann, Eitam Weiss, Avshalom Shalom

**Affiliations:** 1 Department of Plastic Surgery, Meir Medical Center, Tchernichovsky St. 59, 4428164 Kfar Saba, Israel; 2 Affiliated with the School of Medicine, Faculty of Medical and Health Sciences, Tel Aviv University, Tel Aviv, Israel

**Keywords:** Abdominal etching, Abdominal musculature, Body contouring, Liposuction

## Abstract

**Background:**

The abdomen is the aesthetic and physical center of the body. Abdominal etching is used to enhance the appearance of the abdominal musculature. Body contouring and abdominal etching are popular among both men and women, and these procedures have been shown to result in high patient satisfaction and are considered safe. The aim of this study was to describe a novel classification for abdominal etching based on the senior author’s technique and experience.

**Methods:**

This single surgeon, nonrandomized, retrospective study was conducted from December 2016 to September 2022. Patients were classified into 4 groups based on their body habitus, abdominal skin pinch test and skin quality, and the surgical plan was tailored accordingly.

**Results:**

Sixty-two patients (42 male) with an average age of 36 years underwent abdominal etching during the study period. Subgrouping included 4 (6.45%) in Class 1, 22 (35.5%) in Class 2, 32 (51.6%) in Class 3 and 4 (6.45%) in Class 4. The most common complication was seroma. Concomitant procedures included silicone implants to the pectoral region (male), fat injection to the buttocks, breast reduction and mastopexy and treatment of post-liposuction irregularities.

**Conclusions:**

The abdominal etching technique is safe and reproducible. It has demonstrated long-lasting results and high patient satisfaction. Our classification of patients will enable surgeons to better understand the problem presented and provide aesthetic and efficient results. Use of these guidelines and tailoring treatment options will further improve patient and physician satisfaction.

**Level of Evidence IV:**

This journal requires that authors assign a level of evidence to each article. For a full description of these Evidence-Based Medicine ratings, please refer to the Table of Contents or the online Instructions to Authors  www.springer.com/00266

## Introduction

The abdomen is the aesthetic and physical center of the body with the umbilicus located in the focal point. As outlined hundreds of years ago in the Vitruvian masterpiece of Leonardo de Vinci, the distances measured from the distal parts of the upper and lower limbs to the umbilicus are of equal length. This is also in accordance with the embryologic “radial streaming” of development from the umbilical cord as the center of the body.

The “six pack” is a term used to refer to the subunits of the rectus muscle. These muscular bellies are the result of horizontal tendinous intersections separated in the vertical plane by the linea alba and bordered laterally by the linea semilunaris. The term “six pack” is a common misnomer as the number of units varies among individuals. In many people’s view, a muscular appearance imparts the impression of a healthy, athletic person and is considered by some to be more sexually attractive.

As stated by Husain et al., although men and women strive to achieve the desired features of the abdominal wall, including well-defined abdominal musculature and a six pack in men, it is not always possible to attain this solely through exercise and lifestyle modifications. However, surgery can achieve this goal [1].

First described by Mentz et al., abdominal etching is used to enhance the appearance of the abdominal musculature [2]. High-definition liposuction along with abdominal sculpturing introduced new techniques aimed at improving the appearance of the abdominal region and has shown improved outcomes [3]. Body contouring and abdominal etching have gained popularity in the last decade among both men and women [4–6]. High-definition liposuction has been shown to result in increased patient satisfaction and is considered safe [1, 4, 5, 7, 8]. Body contouring includes selective liposuction which may be augmented by fat grafting to enhance the anatomical appearance [9, 10]. The increased interest in body contouring is in part due to the influence of social media and the growing awareness of self-image, with an athletic body and defined muscular appearance considered to be the “ideal” [1, 11–13]. This trend can also be attributed to the growing popularity of fitness training and exercise among the younger population [10].

This study describes a novel classification system for abdominal etching based on the senior author’s technique, in a case series of 62 consecutive patients.

## Materials and Methods

### Study Design

This single surgeon, nonrandomized, retrospective study was conducted from December 2016 to September 2022. A total of 62 patients were eligible for abdominal etching. Data regarding age, gender, previous abdominal surgery (hernia repair, appendectomy, etc.), height, weight, and body mass index (BMI) were collected.

Exclusion criteria were uncontrolled diabetes mellitus, patients with American Society of Anesthesiologists physical status classification ≥ 3, pregnancy, dysmorphic syndromes and patients with a BMI > 30 kg/m^2^.

All patients were documented preoperatively, and 1 week 1, 3 and 12 months and at additional postoperative follow-up visits, in a standardized manner using a Canon EOS 65 camera (Canon Inc., Huntington, NY), at a predetermined patient–camera distance of 2 meters. Surgical outcomes were assessed by patients and the physician at 6 months using a 1-10 Likert-type scale (range from 1-very poor to 10-excellent result). Statistical analysis, including trends of satisfaction, rates and comparisons of means using the t-test, was conducted for the entire cohort and for various subgroups.

### Patient Classification

Patients were divided into 4 groups based on their body habitus, pinch test of abdominal skin and other planned surgical sites, and skin quality, as follows:

Class I Pinch test < 1 cm, with good skin quality.
Class II Pinch test of 1–2 cm, with good skin quality.
Class III Pinch test of 2–3 cm, with good skin quality.
Class IV Pinch test > 3 cm and bad skin quality (striae, stretch marks, extreme weight changes).

It should be noted that BMI was not used to classify patients seeking abdominal etching surgery. This is because many of these patients exercise regularly, have a high muscle-to-fat ratio, a mesomorph body habitus, and some use anabolic steroid supplements.

All patients provided written informed consent. After taking a thorough medical history, a physical examination and analysis of the abdomen were conducted. It should be noted that we use a skin fold caliper to measure the skin pinch, as this is a more reliable tool.

Primary surgical procedures were conducted under general anesthesia, and in some secondary interventions local anesthesia was used. All procedures were documented, including the technique selected, the amount of lipoaspirate and any complications. In cases of fat grafting, the amount of fat injected was also recorded. Patients were followed up, and photographs were taken at 1 week, 1, 3 and 12 months postoperatively and at additional follow-up visits. All data were collected on Microsoft Excel (Redmond, WA) and analyzed for descriptive characteristics.

## Surgical Technique

### Preoperative Marking

Areas are marked with the patient in the standing position. Patients are also asked to position the height they normally wear their underwear or swimsuit to mark the lowest points for incisions for cannula placement. The standing position is preferred to prevent incorrect positioning of the tendon intersections due to the natural upward motion of the skin when supine. The oblique muscles, semilunar lines, linea alba, tendon intersections, inguinal fold and the inframammary lines are also marked. Flank fat depositions are also addressed and marked as a topographic map.

### Intraoperative Procedure

In cases that require significant liposuction of the flanks, the procedure is started with the patient in the prone position. After the patient is prepared and draped, an 11 blade is used to make two 5 mm incisions on the paramedian part of the back, asymmetrically (to prevent liposuction stigmata). Tumescent solution (1000 ml of 0.9% normal saline, 1 ml of 1:1000 adrenaline, 20 mg 2% lidocaine) is infiltrated subcutaneously using a rotating basket 5-mm cannula to the areas to be suctioned. Tranexamic acid (hexakaprone) has been used in the last year and can be added to the solution at the surgeon’s discretion. After waiting 10 mins, power-assisted liposuction (MicroAire, Inc, Charlottesville, VA) is commenced to the flank regions using a 4-mm, one-sided, multi-hole Mercedes cannula. Liposuction is continued until minimal fat remains and equalization is achieved using the 5-mm basket cannula without suction. All aspirated fat is collected in a sterile canister. Incision edges are approximated using a Vicryl rapid 5/0 suture (Ethicon, Somerville, NJ) to prevent buildup of seroma.

The patient is then rotated to the supine position. Three to five additional incisions are made below the underwear line; two to address the semilunar lines, one for the midline and when necessary two more to further address the flanks, laterally. To address the transverse lines, a single incision is made in the umbilical region (upper or lower part depending on the location of the transverse line), another for the first transverse line above the umbilicus and one for the following upper line. Incisions are made adjacent to the semilunar transverse junction, asymmetrically.

The following surgical steps depend on the patient classification (described in Methods, *Patient classification*).

**Fig. 1:**
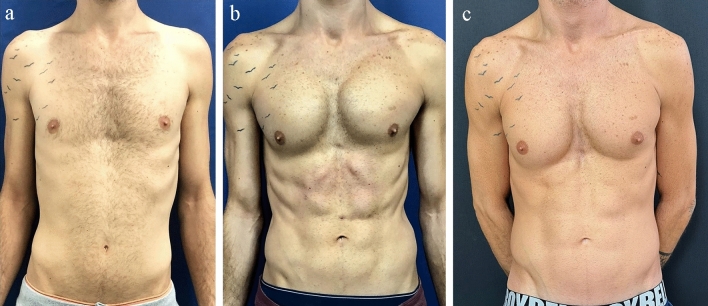
A 27-year-old male classified as Class 1 (thin) who underwent liposuction of 120 cc of fat from the flanks, linea alba and tendinous intersection combined with fat injections of 20 ml to 6 muscular bellies. Pectoral 300-cc silicone implants were added to the chest through an axillary incision, **a** before surgery, **b** 1 year after surgery and **c** 6 years after surgery
Class II Pinch test of 1–2 cm with good skin quality. The same technique as described for Class I is performed, with selective liposuction for definition. Fat injection is unnecessary, as the native fat deposition is thick enough to mimic the rectus abdominis muscle bellies. These are the ideal candidates for abdominal liposuction (Fig. 2a) before surgery, (Fig. 2b) 1 year after surgery, (Fig. 2c) 6 years after surgery.

Class I Pinch test < 1 cm with good skin quality, an additional 1000 ml of tumescent solution (as described above) is injected into the abdomen. When required, aggressive liposuction is performed in the flank regions up to the semilunar lines, leaving minimal fat in the superficial plane and skin as thin as a full thickness skin graft. The same aggressive liposuction is performed along the linea alba and above the tendinous intersections. This is done through the umbilical incision and through another incision made at the upper intersections. Following liposuction, equalization is performed using a 5-mm basket cannula without suctioning. Since these patients are very slim and augmentation of the rectus abdominis muscle is desired, about 10–20 cc of fat is injected subcutaneously above each muscle belly using a 1-mm cannula. No fat is injected into the muscle. The fat is prepared by decantation in a 50-cc syringe, removal of the liquid fraction and transfer into a 10-cc luer lock syringe. The final definition of the inguinal ligamentous lines, semilunar lines and tendinous intersections are achieved by another passage of the 5-mm basket cannula over these lines with suctioning (Fig. 1a) before surgery, (Fig. 1b) 1 year after surgery and (Fig. 1c) 6 years after surgery.

**Fig. 2:**
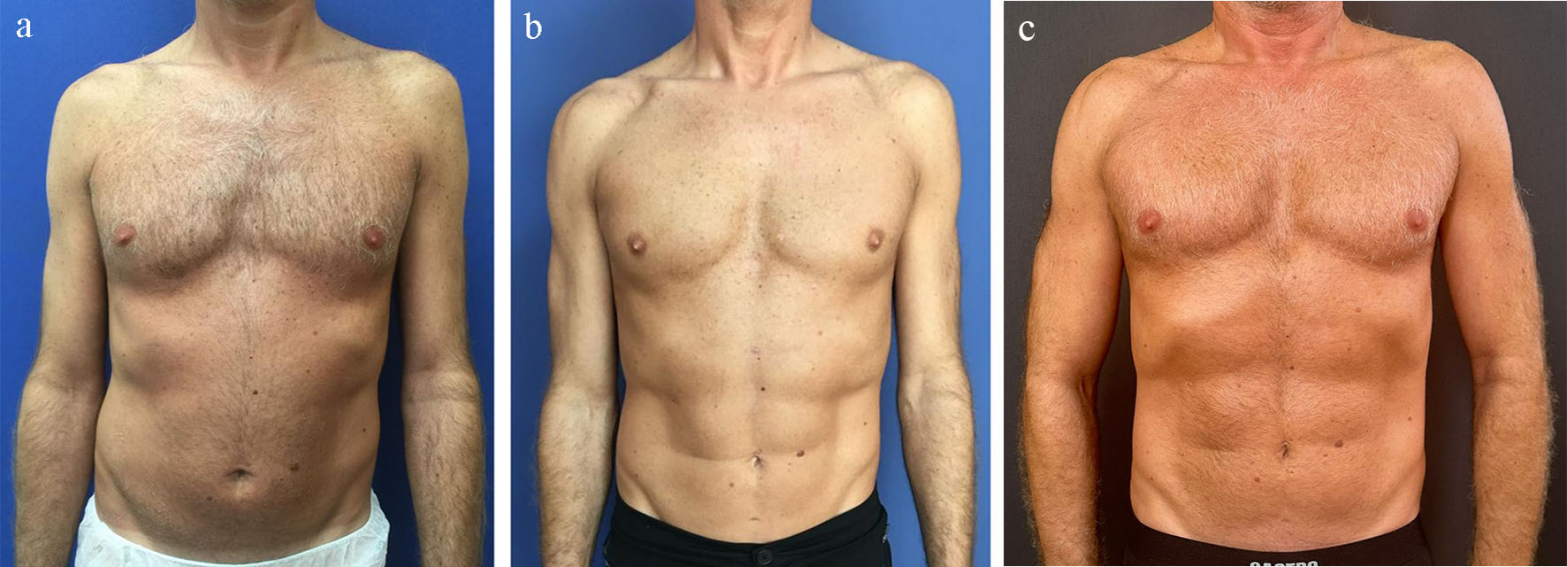
A 42-year-old male classified as Class 2 (athletic) who underwent liposuction of flanks, linea alba and tendinous intersection. A total of 800 cc were liposuctioned and 65 ml of fat was grafted to each pectoral region **a** before surgery, **b** 1 year after surgery, **c** 6 years after surgery
Class III Pinch test of 2–3 cm with good skin quality. Standard, broad liposuction from the lower abdomen is performed first to thin out the abdomen and prevent an inadequate or iatrogenic appearance. The liposuction is commenced until a skin pinch of about 1.5 cm is achieved. The liposuction brings the Class III patient nearer to Class II to enable selective etching of the abdominal musculature. The same technique as described for Classes I and II is then performed. Fat is not injected (Fig. 3a) before surgery, (Fig. 2b) 1 year after surgery, (Fig. 2c) 3 years after surgery. (Fig. 4a) before surgery, (Fig. 4b) 1 year after surgery).

**Fig. 3:**
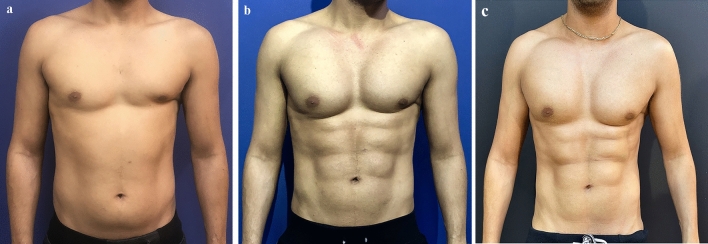
A 27-year-old male classified as Class 3 (overweight) who underwent liposuction of 400 cc of fat from the flanks, linea alba, tendinous intersection and lower abdomen combined with fat injections of 40 ml to 6 muscular bellies. Pectoral 300-cc silicone implants to the chest were also added through an axillary incision. The patient underwent an open chest intervention as an infant for congenital heart disease. **a** before surgery, **b** 1 year after surgery, **c** 3 years after surgery

**Fig. 4:**
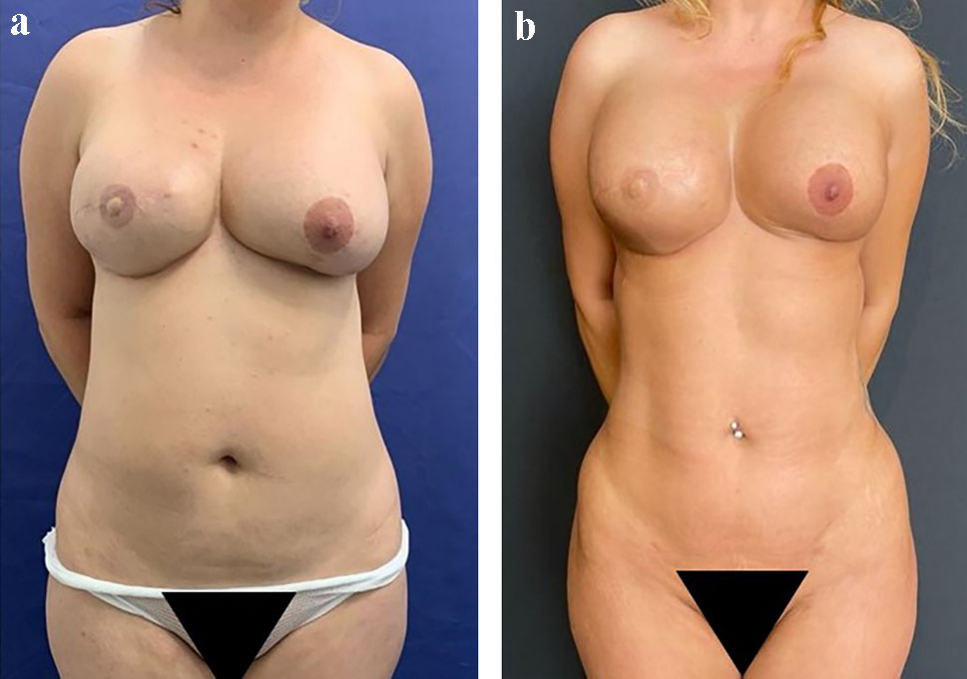
A 35-year-old female classified as Class 3 (overweight) who underwent liposuction of the flanks, linea alba and lower abdomen. A total of 1600 were liposuctioned from all regions. She also had 240 cc of fat injected to each side of the buttocks. The patient had a previous left breast reconstruction and did not desire any further breast intervention. **a** before surgery, **b** 1 year after surgery
Class IV Pinch test > 3 cm and poor skin quality (striae, stretch marks, extreme weight changes). Previous experience with this subset of patients included standard, broad liposuction. Selective etching of the abdominal musculature was minimally applicable to these patients and fat is not injected. Results for these patients have been unsatisfactory for both patient and surgeon; we therefore prefer to refrain from operating on this class of patients.

**Fig. 5:**
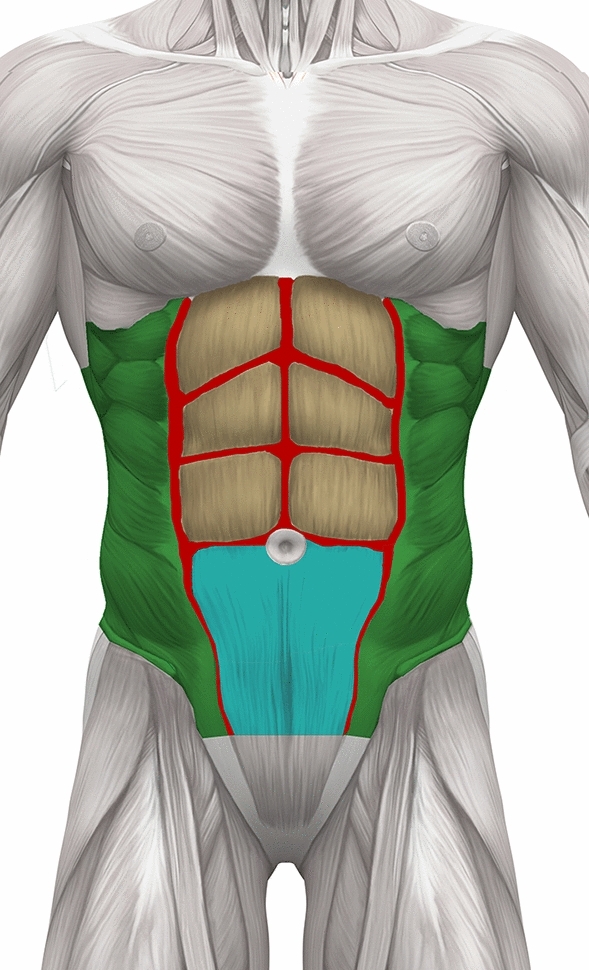
Illustration of the anatomical areas treated in abdominal etching according to the classification system. Green indicates areas where aggressive liposuction is performed in the flank regions up to the semilunar lines, leaving minimal fat in the superficial plane (Classes 1–3). Red denotes lines of aggressive liposuction along the linea alba, above the tendinous intersections and along the semilunar lines (Classes 1–3). Tan is areas for subcutaneous fat injection above each muscle belly. Fat is not injected into the muscle (Class 1). Blue is the lower abdominal area, specifically the inguinal region which undergoes liposuction to enhance the contour

Figure 5 depicts the areas treated with the described techniques.

### Postoperative Care

**Fig. 6:**
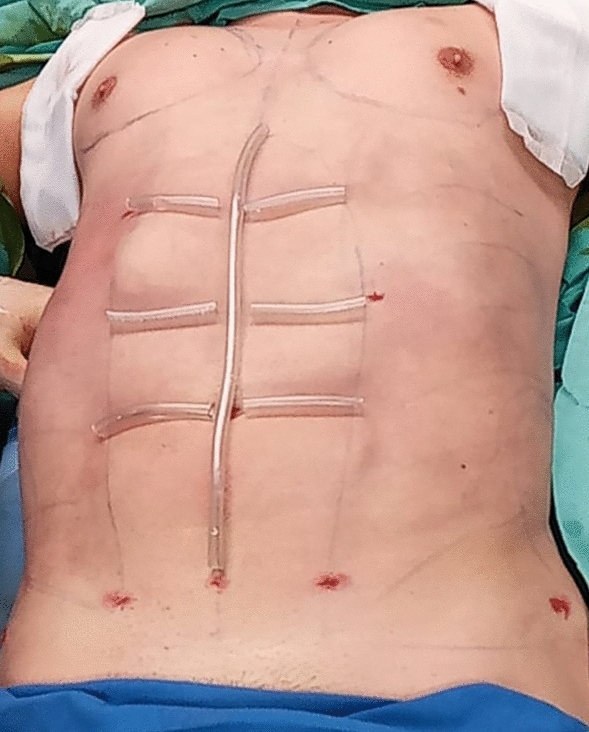
Soft silicone tubing placed over the etched areas and then taped. These are removed on postoperative day 7. The liposuction holes can be seen

After completion of the operation, the abdomen is cleaned with a benzene-based liquid or acetone to dry the skin. Polyurethane adhesive spray (Opsite™, Smith and Nephew, Belgium) is then applied to the newly etched lines and a soft silicone tube (the tumescent tube is preferred) is cut and taped in place for 7 days, using Micropore tape (3M Health Care, St. Paul, MN) in the linea alba and tendinous intersections, creating an elevated ridge on both sides of the groove (Fig. 6). An abdominal binder or compression garment is worn for 6 weeks, preferably continuously during the first 2 weeks and for most of the day afterward.

## Results

Sixty-two patients (42 males and 20 females) underwent abdominal etching by the senior author during the study period. The average age was 36 years (range 20–52), with an average BMI of 22.3 kg/m^2^ (range 16.8–29.4). There were 4 patients (6.45%) in Class 1, 22 (35.5%) in Class 2, 32 (51.6%) in Class 3 and 4 (6.45%) in Class 4 (further described in Table 1).

**Table 1:** Patient characteristics and demographics grouped by classification of body habitus, skin quality and pinch test

Characteristic	Class 1 *N*=4 (%)	Class 2 *N*=22 (%)	Class 3 *N*=32 (%)	Class 4 *N*=4 (%)
*Sex*				
Male Female	2 (50) 2 (50)	18 (82) 4 (18)	20 (62.5) 12 (37.5)	2 (50) 2 (50)
Mean age, years	33.5	33.9	37	38.8
Mean BMI (kg/m^2^)	18.05	21.81	23.34	24.22

The most common complication was formation of seroma. Although this occurred in several patients, none required further intervention and all resolved completely. One case of skin necrosis occurred on the left flank of a female patient. Conservative management was employed until complete healing and scars were later treated with steroid injections. A second liposuction was requested in 20 cases (30%) to enhance results. Concomitant procedures included 4 silicone implants to the pectoral region (male), 4 fat injections to the buttocks, 1 breast reduction and mastopexy and 1 post-liposuction irregularity treated with subincision and fat injection. All patients were discharged the same day of the procedure.

The mean satisfaction rate for the entire cohort was 7.56 (range 4–10), and the surgeon’s overall satisfaction rate was 7.57 (range 4–10). Subgroup analysis showed that patients in Class 2 (athletic) were most satisfied with their results (7.6) and Class 4 (obese) were the least satisfied (7.25). Further subgroup analysis showed that the mean satisfaction rate was slightly higher for men than women (7.78 vs. 7.05, *p*=*NS*). Furthermore, patients who had an adjunct procedure during the abdominal etching had a mean satisfaction rate of 7.77 and those who underwent a secondary procedure had a mean satisfaction rate of 8.21 (*p*=*NS*).

All patients had at least 1 year of follow-up and several returned annually for follow-up visits (Figs. 1–4).

## Discussion

This study found that high-definition liposuction and abdominal etching done according to the novel classification system, as described in 62 consecutive patients, were shown to be safe and effective. Satisfaction with results of the technique was high both among patients and the surgeon. The most common complication was seroma in the liposuctioned areas; yet, all resolved with conservative treatment. It is imperative to explain to patients that seroma formation can be expected [14] and that it will resolve in most cases either following a single aspiration or with watchful waiting. Danilla et. al also reviewed complications of high-definition liposuction and treatment options. In their study, about 30% of cases had seroma formation and all responded to percutaneous aspiration [14]. The high rates of seroma in high-definition liposuction and body contouring can be secondary to aggressive liposuction and damage to superficial lymphatic tissues. The use of energy-based liposuction can also increase production of seroma and has been related to skin hyperpigmentation [1, 3, 14–16]. Several authors addressed the buildup of seroma following liposuction with various adjunct modalities suggested to reduce the formation of seroma, including the use of drains and pressure garments [1, 14, 17].

Our technique uses power-assisted liposuction and has not encountered any instance of skin hyperpigmentation. We do not place drains and abdominal binders are fitted in all cases. When considering seroma formation in our patient cohort, the majority of cases resolved spontaneously and those that required percutaneous aspiration did not have any further complications following the intervention. Skin necrosis is a dreaded complication and can be caused by aggressive superficial liposuctioning or the use of energy-based modalities [3, 14, 15]. Meticulous technique can prevent this complication. In the one case described in our study, the skin necrosis was on a minor surface area, was treated conservatively, and healed well.

Thirty percent of patients returned to request a second liposuction to further enhance the results already achieved. It must be stated that these were small “touch-ups” and enhancements that included minor liposuction and grafting revisions. We believe that it is imperative to consult this subset of patients more meticulously regarding the expected result and their expectations early in the process, to decrease the number of secondary procedures.

Most patients referred for abdominal etching in our cohort followed a healthy lifestyle and exercised regularly. As in other studies [5, 7], we believe that patients who opt to undergo this procedure will maintain their lifestyle and the procedure might have a positive effect on this. Our patients also reported that following the surgery, they invest more time into maintaining their appearance. In most cases, female patients did not request extreme definition and were satisfied with moderate definition. Patient and surgeon satisfaction rates were around 7.5, and the highest satisfaction rates were in Class II patients. Although these might seem low to the observer, it should be kept in mind that our patients are a unique subset. These patients tend to be more demanding and more conscious about their appearance. As for the surgeon, aesthetic surgery is a demanding field and surgeons at times tend to be overly judgmental of their results, always aspiring higher.

The abdominal contour and appearance are affected by the anatomical components of the abdominal wall, including fat deposition and muscular mass, body habitus, skin laxity, age and genetics. Various techniques for body contouring can be offered to patients seeking a more defined and athletic look. Every patient is unique, and the procedure offered should be tailored to his/her expectations and in accordance with acceptable esthetic surgical guidelines. Therefore, we categorized patients by classes to better match the surgical approach in accordance with the characteristics of each class.

The introduction of high-definition liposuction and abdominal etching have enabled plastic surgeons to offer enhanced and refined results tailored to each patient [3, 18]. Like many new techniques and modalities, this approach has also led to the introduction of complications, indications and limitations [14, 15]. Patients’ expectations are rising, and this is in part due to exposure to social media and the advances in results that can be offered to patients. Hoyos et al. reviewed the technique, describing the advances that arose from liposculpture, 4D lipo, muscle dynamics, and ever evolving techniques aiming to achieve defined and enhanced body contours while maintaining patient safety [18]. They concluded that although current results offered to patients may be considered by some to be excellent, it is our duty as plastic surgeons to keep analyzing and studying our techniques and outcomes to further advance the field and maintain patient safety [18].

Another emerging technology employed for body contouring and abdominal etching is the use of Renuvion radiofrequency-helium plasma energy (Apyx Medical, Clearwater, FL). Brummund and Husain described their experience with this modality for abdominal etching and showed that it was safe, with high patient satisfaction [19].

In a review of past and present approaches to abdominal etching, Agochukwu-Nwubah and Mentz [20] described different techniques, including the full or modified approach and the refinements the procedure has undergone in recent years. Saad et al. [4] proposed a classification for the level of etching and definition based on the amount of superficial fat liposculpting.

Viaro [5] classified the extent of improvement in the surgical procedure in female patients based on patient and surgeon observations. According to these surgical approaches and classifications, several studies have tried to answer the question of “what is the ideal abdomen?” [11, 12].

As described previously, the importance of patient selection for this surgery cannot be over-emphasized [13]. Based on our progressive experience, skin quality is the most important factor in predicting surgical outcomes. Adequate evaluation of skin has a learning curve that is based on experience and includes parameters such as dermal thickness, striae, wrinkles, turgor and elasticity. The ability to adequately assess a patients’ skin quality requires a learning curve; yet, this can be mastered by meticulous attention to patients’ skin characteristics and constant evaluation of postsurgical results. In our study, the surgeon relied on experience gained over time to assess skin quality, along with observation, history taking and the simple clinical skin pinch test.

Patient history including post-bariatric surgery, changes in weight, twin pregnancies or significant weight gain during pregnancy all has detrimental effects on skin quality. The presence of striae warrants further cautious examination and, in some cases, should be considered a relative contraindication to abdominal contouring surgery. Previous experience with Class IV patients has been unsatisfactory. Therefore, it is recommended to refrain from performing the described technique in individuals with these characteristics.

Hoyos et al. [6] sub-classified patients according to their body type (endomorph, ectomorph and mesomorph). In our cohort, we also noticed that patients in class 1 were thin and class 2 were mostly athletic. In accordance with Hoyos et al. [6], our classification also relies on body type.

The previous classifications of patients discussed above rely on body type, amount of lipoaspirate, fat injected or surgical improvements in body contour. Through the experience gained and analysis of our new classification system in this study, we introduce the use of a pinch test and meticulous analysis of skin quality. The use of the pinch test is an excellent marker for subcutaneous fat deposition.

We also rely on skin quality, which in our experience is the most important factor predictive of excellent surgical outcomes. We believe that this expansion of the subclassification of patients presenting for high-definition liposuction and abdominal etching can further assist the surgeon in selecting the right technique and tailoring it perfectly for every patient. Classifications should be used to guide surgeons to select the correct modalities and procedures that can be offered to various patients. The success of these is dependent on cautious and meticulous patient analysis.

The patient classification system presented in this study broadens the tools that can be used by surgeons performing abdominal etching and will help surgeons categorize their patients according to simple physical and clinical features. By using these guidelines and classifications, together with surgery personalized for every patient, our results improve and our techniques continue to evolve. Accordingly, the outcomes are more predictable and consistent.

As with all studies, this study also had several limitations. The number of cases included in this study was relatively small, and all cases were performed by a single surgeon. Although reviewing a single surgeon’s work might lead to observer bias, this also has the advantage of consistency in technique. To limit the potential for bias, all authors, both residents and expert plastic surgeons reviewed photographs of the patients included in the study. All surgeries were conducted by a single surgeon; yet, as stated this may lead to consistency in approach and technique and permit learning and enhancement of the technique. The use of the pinch test has its limitations due to the inherent changes in skin and subcutaneous fat thickness in various anatomical regions, which can also vary between genders. Yet, when evaluated based on various areas to be treated, use of a pinch test, experience gained and other patient variables help the surgeon classify the patient and plan the procedure best suited to the individual. Another limitation was the use of a questionnaire for assessing patient and surgeon satisfaction that was not validated. Future studies should include the use of a validated satisfaction questionnaire.

## Conclusions

The technique and classification system for abdominal etching presented here have proved to be safe and reproducible, with highly satisfactory results. Classifying patients will enable surgeons to better understand their characteristics, which will contribute to and provide effective and aesthetic results. Use of these classification guidelines for surgery and tailoring treatment options will further improve patient and physician satisfaction.
